# Congenital Spigelian Hernia and Cryptorchidism: Another Case of New Syndrome

**Published:** 2013-10-23

**Authors:** Dhiraj Parihar, Yogender Singh Kadian, Preeti Raikwar, Kamal Nain Rattan

**Affiliations:** Department of Surgery BPS Government College for Women Khanpur Kalan Sonepat; Department of Paediatric Surgery PT B D Sharma PGIMS Rohtak (Haryana); Department of Paediatric Medicine BPS Government College for Women Khanpur Kalan Sonepat; Department of Paediatric Surgery PT B D Sharma PGIMS Rohtak (Haryana)

**Keywords:** Spigelian hernia, Cryptorchidism, Inguinal canal, Agenesis

## Abstract

Spigelian hernia (SH) is rarely seen in pediatric age group and is usually associated with cryptorchidism on the same side; termed as a syndromic association of the defect in the Spigelian fascia and absence of gubernaculum and inguinal canal. The absence of the inguinal canal has surgical implication as to placement of the undescended testis into the scrotum. A 3-month-old baby presented with spigelian hernia and ipsilateral impalpable testis. The spigelian hernia was repaired and undescended testis which was present in abdominal wall layers was brought to scrotum with cord structures anterior to external oblique muscle.

## INTRODUCTION

SH is rare in children with only 59 pediatric cases reported up until 2012 since its first description in 1935.[1-4] The anatomical basis of SH is weakness in the SH belt arising from a transversus abdominis aponeurosis between the lateral edge of the rectus sheath and the semilunar line that stretches from the ninth rib to the pubic tubercle. Whereas adult SHs are considered to be acquired because of trauma or increased intra-abdominal pressure, pediatric cases are suspected to be congenital.[1,5] Among male infants with SHs, 75% are associated with cryptorchidism.[6] The association of ipsilateral undescended testis and SH is being debated whether it is a cause or effect relationship. Raveenthiran suggested that a defect in the Spigelian fascia combined with ipsilateral cryptorchidism, may be a part of new syndrome.[7] Rushfeldt and others suggested that the additional lack of a gubernaculum and inguinal canal in similar cases, may be considered as two additional and closely connected elements of this new syndrome.[2] We have managed a 3 months old male child who presented with SH and ipsilateral undescended testis along with all the features of this new syndrome.

## CASE REPORT

A 3-month-old male baby was admitted with a swelling on the right side of abdomen and absence of testis on the same side. The swelling increased in size on crying. The swelling occupied right lower quadrant of the abdomen and was non-tender. On genital examination, the left testis was normally descended and right one was impalpable in scrotal pouch and inguinal area (Fig. 1A). Ultrasonography of the swelling revealed an echogenic tissue consistent with testis in the layers of abdominal wall along with small gut loops echoes. At operation a defect found on the lateral border of rectus abdominis muscle around the arcuate line with a hernial sac containing testis without any inguinal canal and gubernaculum (Fig. 1B).The sac was opened and the testis was separated from the sac. The defect in the abdominal wall was repaired by absorbable sutures and testis placed in the right sub-dartos pouch by placing the cord structures anterior to the external oblique muscle (subcutaneous route). In the post operative period child remained well and discharged home after 3 days.

**Figure:**
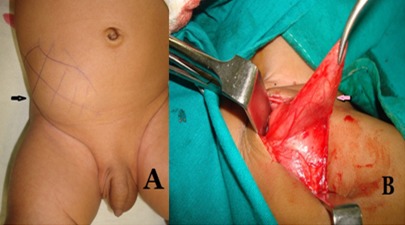
Figure 1: (A) Clinical photograph of the child showing swelling on the right side of abdomen and empty right hemiscrotum. (B) Operative photograph showing the hernia sac containing testis.

## DISCUSSION

Cryptorchidism in children presenting with SH is considered to be a congenital condition. There has been considerable speculation and controversy regarding the possible mechanism of cryptorchidism in SH. According to the general viewpoint, SH is the primary defect and the undescended testis takes the path of least resistance to descend and lie in the hernial sac accounting for this association.[6,8,9] Raveenthiran speculated an ectopic location of the testis is the primary abnormality, and it leads to formation of SH by dragging a peritoneal sac along with it.[7] Rushfeldt et al believed in failure of development of gubernaculum as an inciting event; [2] thus inguinal canal could not develop and the testis remain in their intra-abdominal position.[2,10]

Ultrasonography helps in assessing the size of the defect and contents of the hernia sac. The management of this anomaly includes repair of the hernia and scrotal placement of the undescended testis. While undertaking surgical repair of SH, one should look for an undescended testis, and expect it to be in the hernia sac. Furthermore, since an inguinal canal may not be found, one should be prepared to perform an orchidopexy either via a subcutaneous route or a new internal ring may be created through the abdominal wall medial to the inferior epigastric vessels and just lateral to the pubic tubercle.[4] However one should be careful in orchidopexy by creating neo internal ring as testicular atrophy has been reported in one patient.[4] The possible reported explanations for such an outcome include vascular damage, tension and compression of the cord and scrotal infection.

## Footnotes

**Source of Support:** Nil

**Conflict of Interest:** None declared

